# Effect of Bailing capsule complicated with low-calcium peritoneal dialysis solution on residual renal function and microinflammatory status in peritoneal dialysis patients with chronic renal failure

**DOI:** 10.3389/fendo.2025.1561062

**Published:** 2025-10-07

**Authors:** Xiancheng Li, Yafeng Zhao, Xiaoyong Yu, Shufei Wang, Kai Qu, Yu Wang

**Affiliations:** Second Department of Nephrology, Shaanxi Provincial Hospital of Chinese Medicine, Xi’an, Shaanxi, China

**Keywords:** chronic renal failure, peritoneal dialysis, Bailing capsule, low-calcium peritoneal dialysis solution, residual renal function, microinflammatory state

## Abstract

**Objective:**

This study aimed to ascertain the efficacy of Bailing capsule (BLC) combined with low-calcium peritoneal dialysis solution (PDS) in peritoneal dialysis (PD) patients with chronic renal failure (CRF).

**Methods:**

Ninety-two patients with CRF were randomly divided into a control group (n = 45) and a study group (n = 47). The control group received low-calcium peritoneal dialysis combined with a low-protein diet and compound α-ketoacid tablets, while the study group received BLC in addition to the control group’s treatment. Outcome measures included clinical efficacy, residual renal function (RRF), microinflammatory status, nutritional status, oxidative stress markers, and the incidence of adverse reactions.

**Results:**

The study group demonstrated a significantly higher total effective rate compared to the control group (*P* < 0.05). Post-treatment, the study group exhibited a reduced rate of RRF decline, lower levels of serum tumor necrosis factor-α, C-reactive protein, and interleukin-6, improved serum prealbumin, albumin, transferrin, and hemoglobin levels, reduced serum malondialdehyde levels, and increased serum superoxide dismutase and glutathione peroxidase levels compared to the control group (*P* < 0.05). There was no significant difference in the overall incidence of adverse reactions between the two groups during treatment (*P* > 0.05).

**Conclusion:**

The combination of BLC with low-calcium PDS is effective in PD patients with CRF, demonstrating the ability to slow the decline of RRF, improve microinflammatory status, enhance nutritional and oxidative stress parameters, and maintain a comparable safety profile without increasing the incidence of adverse reactions.

## Introduction

Chronic renal failure (CRF), also referred to as chronic kidney disease (CKD), is defined as a persistent impairment of renal function. It is characterized by abnormally high serum creatinine levels persisting for more than three months or a calculated glomerular filtration rate of less than 60 ml/min/1.73 m^2^. Renal failure represents as a progressive loss of renal function, often necessitating renal replacement therapies such as dialysis or kidney transplantation. When a patient reaches this stage, the condition is classified as end-stage renal disease (ESRD) (1). CRF results from the gradual progression of CKD and requires management through dialysis therapy or kidney transplantation (2). It is a highly morbid condition that, if left untreated, can advance to uremia, causing a variety of complications, including respiratory distress, psychiatric disorders, hypertension, and heart failure. Clinical management of CRF often involves pharmacologic interventions to mitigate its progression and associated complications (3).

In China, *Cordyceps sinensis* (*C. sinensis*) is widely used in the treatment of various kidney diseases. Bailing capsule (BLC), which contains *C. sinensis* as its active ingredient, is an approved treatment for renal, respiratory and immune diseases (4). BLC is a commonly utilized traditional Chinese medicine for managing CKD. A recent study identified 190 common targets between BLC and CKD, suggesting that the pharmacological effects of BLC may involve modulation of inflammatory and immune responses, vascular endothelial injury, cell proliferation, and fibrosis. This suggests that BLC exerts therapeutic effects through multiple pathways and targets, providing a theoretical basis for its clinical application (5). Peritoneal dialysis (PD) is a renal replacement therapy in which a sterile solution is introduced into the peritoneal cavity via a catheter. The peritoneum acts as a semipermeable membrane to facilitate the removal of solutes and water. The solution, enriched with an osmotic agent (most commonly glucose), interacts with the capillaries in the peritoneum to enable diffusive solute transport and osmotic ultrafiltration (6). PD is a viable home-based therapy for renal failure, accounting for 11% of all dialysis treatments globally and 9% of kidney replacement therapies. PD offers several potential advantages over hemodialysis, including simplicity of the technique, lower costs, potentially higher survival rates in the initial years of treatment, fewer dietary restrictions, better preservation of residual renal function (RRF), improved patient satisfaction, better outcomes following renal transplantation, delayed need for vascular access (especially for children), reduced reliance on erythropoietic drugs, and a lower risk of blood-borne viral infections (7). Despite these advantages, few studies have examined the efficacy of BLC combined with PD in the treatment of CRF. Hence, this study focuses on the combination of BLC and low-calcium PD solution (PDS) to evaluate their combined effects on RRF and microinflammatory status in PD patients with CRF.

## Materials and methods

### Ethics statement

The study was approved by the Ethics Committee of Shaanxi Provincial Hospital of Chinese Medicine. All patients and their families signed an informed consent form.

### Participants

The patients with CRF who were admitted to Shaanxi Provincial Hospital of Chinese Medicine between December 2020 and January 2023. The following were the inclusion criteria: patients meeting the diagnostic criteria for CRF (7); patients aged > 18 years; those with indications for PD who had been undergoing PD for more than three months; those having RRF; patients with expected survival > 6 months; those with complete clinical data. The following were the exclusion criteria: patients suffering from other serious renal diseases; those with significant dysfunction or serious injuries to the heart, brain, lungs, or liver; those diagnosed with malignant tumors, autoimmune diseases, or hematological diseases; those with coagulation dysfunction; those suffering from serious psychiatric disorders; those in the period of pregnancy or lactation; and those with known allergies to the drugs used in this study.

### Randomization and blinding

In this clinical study, randomization was employed, with cases randomly assigned to each group. The results of randomization were placed in sealed envelopes, which were consecutively numbered. Upon enrollment, patients received envelopes in the order of their enrollment and were grouped according to the envelope contents. In the waiting list group, there were 47 cases in the study group and 47 in the control group, with a ratio of 1:1. The random sequence was generated by an independent professional statistician not involved in the study using SPSS software (SPSS 24.0, IBM Corp, Armonk, N.Y, USA). Random numbers were stored by fixed, unrelated personnel. Participants were informed that they would randomly receive one intervention after enrollment. Outcome assessors and data statisticians were blinded to group allocation and were responsible for collecting and analyzing data.

### Interventions

The control group underwent PD treatment using a low-calcium PDS. A 10-mm incision was made near the center of the umbilicus to insert a PD catheter. The low-calcium PDS had a calcium ion concentration of 1.25 mmol/L and a glucose concentration of 1.5%-2.5% (8). Both the low-calcium PDS and PD equipment were purchased from Baxter, USA. The dialysis regimen consisted of 2L of dialysis solution per session, with 3–4 exchanges daily at intervals of 4–6 hours, conducted 5 days per week. The dialysis protocol was adjusted based on the results of peritoneal equilibration tests and ultrafiltration measurements. During dialysis, attention was paid to controlling blood pressure and blood sugar, promptly correcting electrolyte and acid-base balance disorders, correcting anemia, and providing a high-quality, low-protein diet (0.6–0.8 g/(kg·d)), low-salt, and low-fat diet. Compound α-ketoacid tablets (Beijing Fresenius Kabi Pharmaceutical Co., Ltd., National Medical Products Administration Approval Number H20041442, specification: 0.63 g×100 tablets) were taken orally with meals, 4 tablets per dose, 3 times a day.

Patients in the study group received BLC in addition to the control group’s treatment. People took BLC orally (Manufacturer: Hangzhou Zhongmei Huadong Medicine Co., Ltd, Hangzhou, China; State Drug Administration Z10910036; Specification: 0.5 g × 42 capsules) at a dose of 2g per session, three times daily (9, 10), with a treatment duration of 8 weeks.

### Outcomes

#### Clinical efficacy

Clinical efficacy was assessed in both groups after 8 weeks of treatment. According to the “Guidelines for Clinical Research of New TCM Drugs” (11), outcomes were classified into three grades: markedly effective (significant relief of clinical symptoms such as fatigue, lumbago, nocturia, and anorexia, with a decrease in serum creatinine and blood urea nitrogen of more than 1/2 from baseline); effective (relief of clinical symptoms with a decrease in serum creatinine and blood urea nitrogen of more than 1/3 from baseline); and ineffective (failure to meet the above criteria). The overall effective rate = (number of markedly effective + effective cases)/total number of cases × 100%.

### RRF

Before treatment and after 8 weeks of treatment, the fasting venous blood (5 mL) was collected from each patient. Serum was separated by centrifugation at 3000 r/min for 5 minutes. Morning urine samples were collected, and 24-hour urine volume was recorded. Urea nitrogen (BUN), serum creatinine (SCr), and urinary creatinine (UCr) were measured using a Beckman Coulter automatic biochemical analyzer (USA). RRF (12) was calculated as [(urinary BUN concentration/serum BUN concentration) × 24-hour urine volume/1440 + (UCr concentration/SCr concentration) × 24-hour urine volume/1440)]/2. Rate of decline in RRF = (RRF before treatment - RRF after treatment)/observation time.

### Microinflammatory state

At the same time points (before treatment and 8 weeks after treatment), 5 mL of fasting venous blood was collected and centrifuged at 3000 r/min for 5 minutes to separate serum. Levels of serum inflammatory markers, including tumor necrosis factor-α (TNF-α), C-reactive protein (CRP), and interleukin-6 (IL-6), were assessed using enzyme-linked immunosorbent assay (13). Reagent kits were purchased from Nanjing Jiancheng Bioengineering Institute.

### Nutritional status

Similarly, 5 mL of fasting venous blood was collected at both time points, centrifuged, and the serum was separated. Nutritional markers, including serum prealbumin (PA), albumin (ALB), transferrin (TRF), and hemoglobin (Hb), were measured using a Hitachi 7180 fully automatic biochemical analyzer (14).

### Oxidative stress markers

Similarly, 5 mL of fasting venous blood was collected at both time points, centrifuged, and serum was separated. The malondialdehyde (MDA) levels were examined by thiobarbituric acid method, superoxide dismutase (SOD) levels were determined by xanthine oxidase method, and glutathione peroxidase (GSH-Px) levels were assayed by colorimetric dithiodinitrobenzoic acid method (15). All reagent kits were purchased from Nanjing Jiancheng Bioengineering Institute.

### Occurrence of adverse reactions

Adverse reactions during the treatment period, including dizziness and headache, nausea and vomiting, skin itching, abdominal pain, bloating, and other symptoms, were recorded for both groups.

### Sample size calculation

The sample size calculation was based on the results of our preliminary study, in which the overall clinical effective rate was 64.44% in the control group and 89.36% in the study group. Using PASS2021 software, we calculated a sample size of 82 to achieve a power of 0.8 and an alpha error of 0.05. Assuming a dropout rate of 15%, 94 patients were recruited for the trial.

### Statistical analysis

SPSS 24.0 software (IBM Corp, Armonk, N.Y, USA) and GraphPad Prism 6.01 software (Graph Pad Inc., La Jolla, CA, USA) were employed for data processing. Measurement data were described as mean ± standard deviation (mean ± SD), and an independent samples t-test was implemented for between-group comparisons and paired samples t-test for within-group comparisons. Numeration data were presented as frequencies and percentages [n (%)], and comparisons between groups were made by utilizing the χ^2^ test or Fisher’s exact test. The significance level was set at *P* < 0.05.

## Results

### Baseline characteristics of study participants

**Figure 1:**
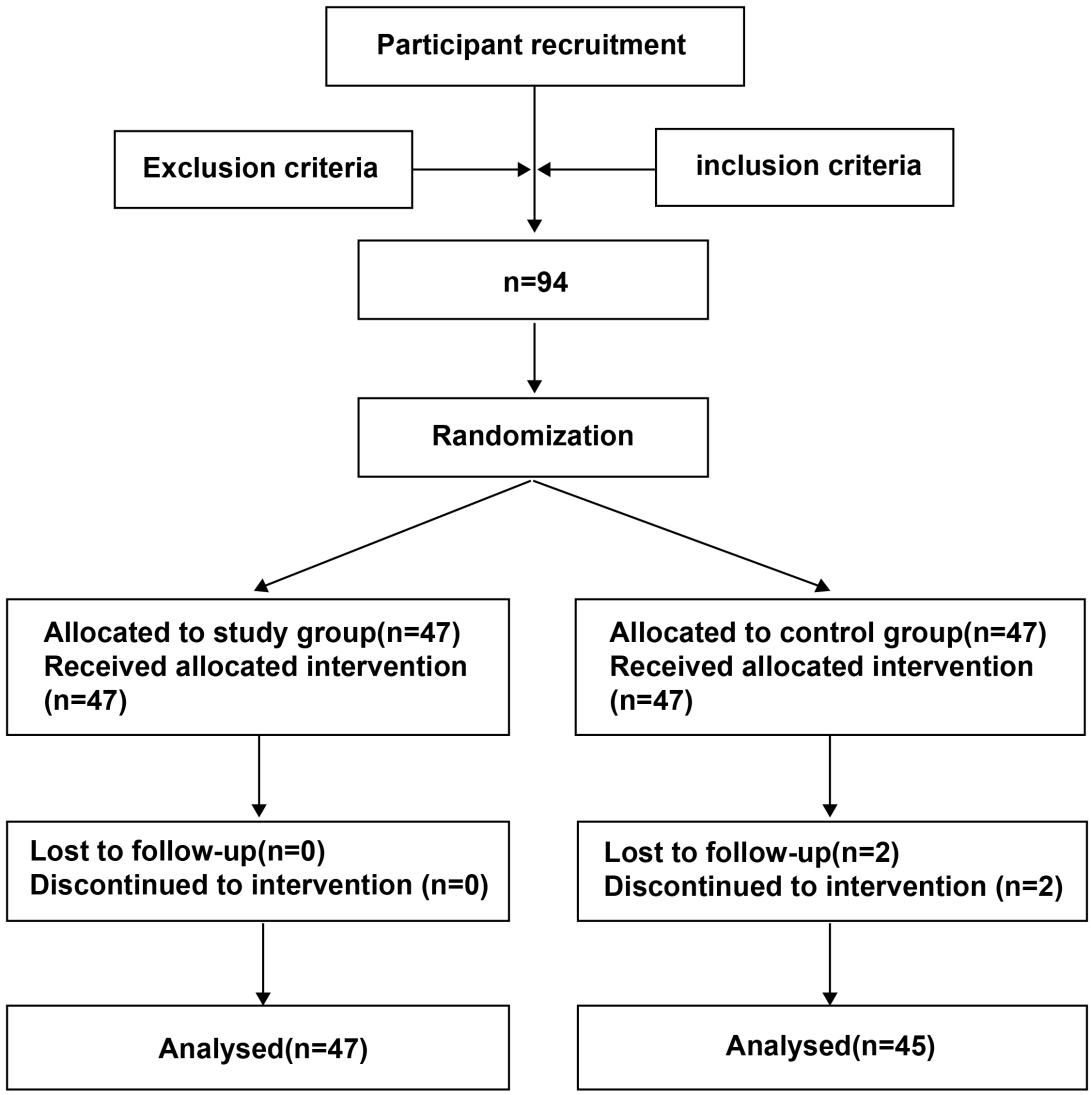
Flowchart of participant recruitment.

After initial screening, 94 individuals met the inclusion criteria and were randomly divided into the control group and the study group, with 47 individuals in each group. Two individuals in the control group withdrew midway. **Figure 1** shows the flowchart of patient inclusion. No statistically significant differences were observed between the two groups in terms of age, gender, body mass index, disease duration, or primary disease (*P* > 0.05), indicating that the two groups were comparable (**Table 1**).

**Table 1:** Comparison of clinical data between the two groups.

Items		The control group (n = 45)	The study group (n = 47)	*P* value
Age (years)		54.16 ± 6.72	54.66 ± 6.91	0.724
Gender	Male	25 (55.56%)	28 (59.57%)	0.697
	Female	20 (44.44%)	19 (40.43%)	
Body mass index (kg/m^2^)		24.68 ± 2.62	24.47 ± 2.60	0.689
Disease duration (years)		3.68 ± 1.42	3.48 ± 1.48	0.521
Primary disease	Chronic Glomerulonephritis	22 (48.89%)	24 (51.06%)	0.980
	Diabetic nephropathy	11 (24.44%)	10 (21.28%)	
	Hypertensive nephropathy	7 (15.56%)	7 (14.89%)	
	Others	5 (11.11%)	6 (12.77%)	

Data as n(%) for categorical variables or mean ± SD for continuous variables.

### Clinical efficacy

Clinical efficacy was observed in both groups after 8 weeks of treatment, and the results showed that the total effective rate in the study group was higher versus that of the control group (*P* < 0.05) (**Table 2**).

**Table 2:** Comparison of clinical efficacy between the two groups.

Efficacy	The control group (n = 45)	The study group (n = 47)	*P* value
Markedly effective	16 (35.55%)	25 (53.19%)	–
Effective	13 (28.89%)	17 (36.17%)	–
Ineffective	16 (35.56%)	5 (10.64%)	–
Total effective rate	29 (64.44%)	42 (89.36%)	0.004

Data as n(%).

### RRF

**Figure 2:**
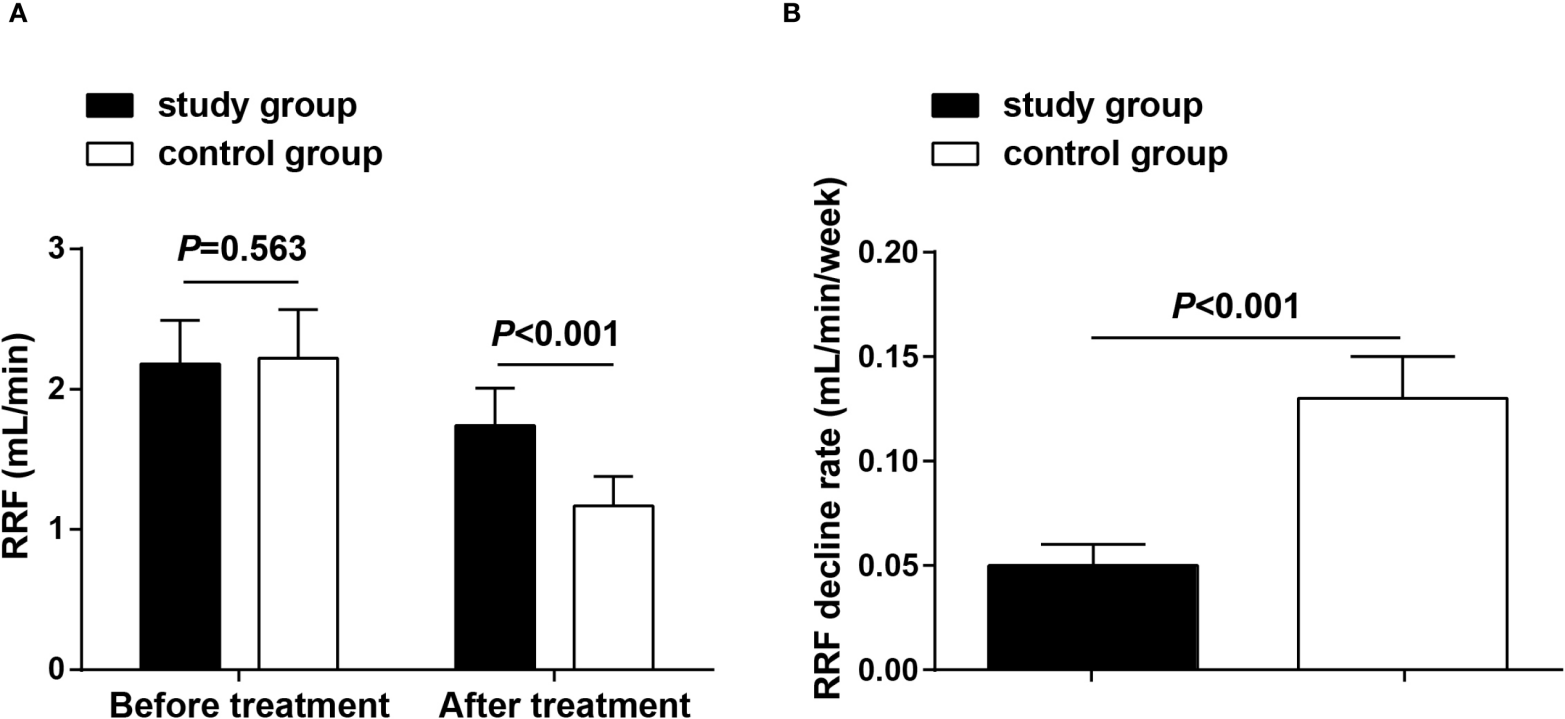
RRF before treatment and after 8 weeks of treatment. **(A)** RRF in both groups before treatment and after 8 weeks of treatment; **(B)** Rate of decline in RRF in both groups after 8 weeks of treatment. Data as mean ± SD. RRF, residual renal function.

RRF was observed before treatment and after 8 weeks of treatment, and the results showed that before treatment, there were no significant differences between the two groups in RRF (*P* > 0.05), demonstrating their comparability; after treatment, both groups showed reduced RRF compared to their baseline levels. Moreover, the decrease in RRF in the study group was less than that in the control group (*P* < 0.05) (**Figure 2**).

### Microinflammatory status

**Figure 3:**
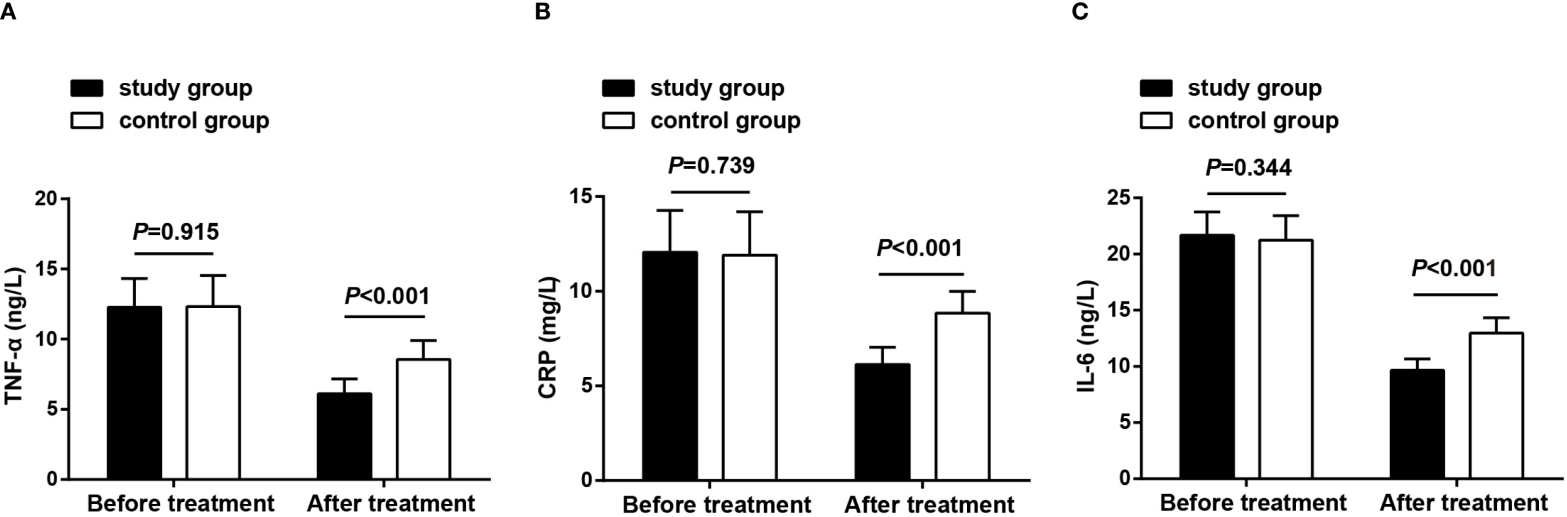
Microinflammatory status before treatment and after 8 weeks of treatment. **(A)** TNF-α levels in both groups before treatment and after 8 weeks of treatment; **(B)** CRP levels in both groups before treatment and after 8 weeks of treatment; **(C)** IL-6 levels in both groups before treatment and after 8 weeks of treatment. Data as mean ± SD. TNF-α, tumor necrosis factor-α; CRP, C-reactive protein; IL-6, interleukin-6.

Observation of inflammatory cytokine levels before treatment and after 8 weeks of treatment revealed that before treatment, the comparison of serum TNF-α, CRP, and IL-6 levels between the two groups presented no significant differences (*P* > 0.05), demonstrating their comparability; after treatment, these inflammatory markers decreased in both groups, with the study group showing lower levels than the control group (*P* < 0.05) (**Figure 3**).

### Nutritional status

**Figure 4:**
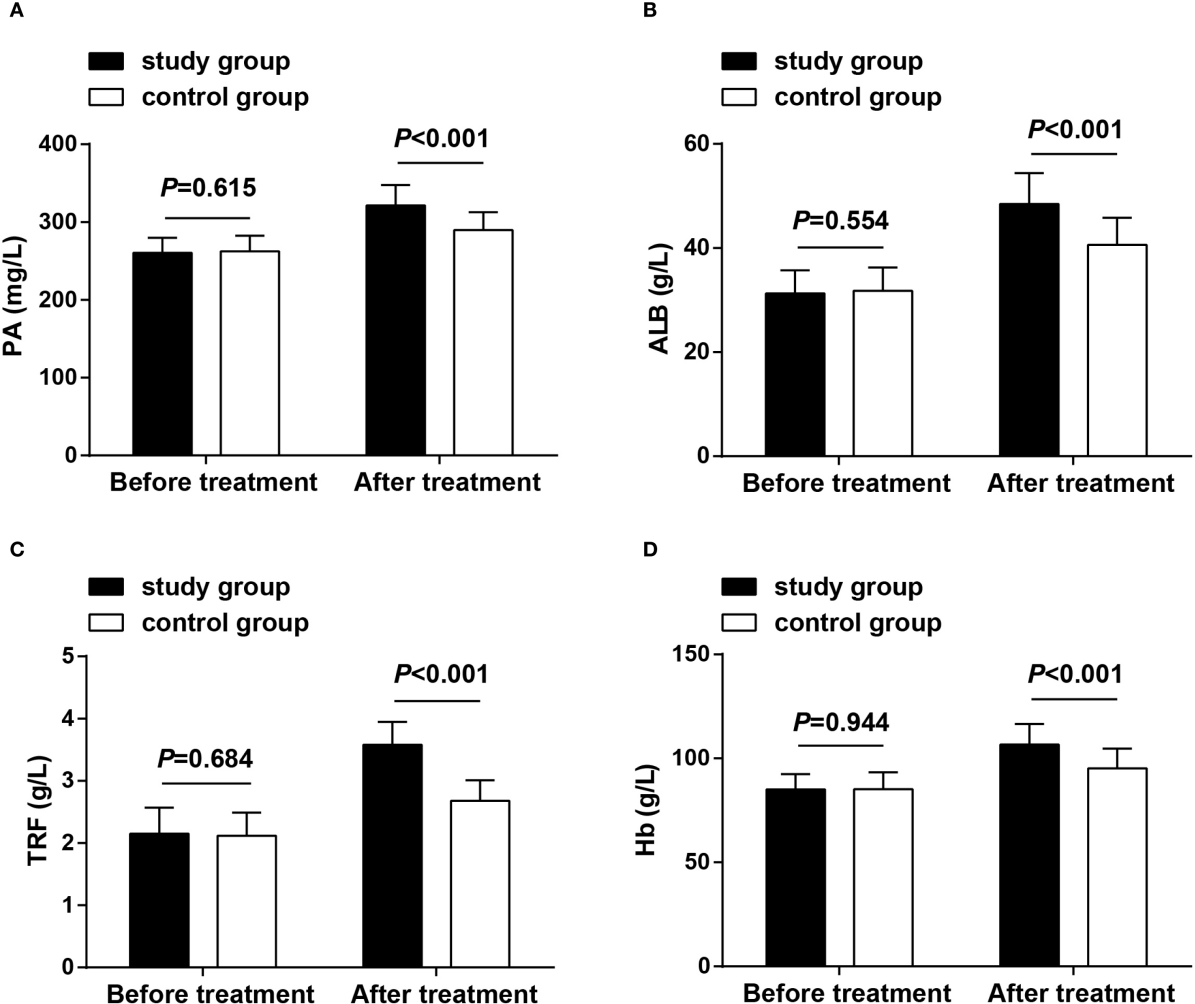
Nutritional status before treatment and after 8 weeks of treatment. **(A)** PA levels in both groups before treatment and after 8 weeks of treatment; **(B)** ALB levels in both groups before treatment and after 8 weeks of treatment; **(C)** TRF levels in both groups before treatment and after 8 weeks of treatment; **(D)** Hb levels in both groups before treatment and after 8 weeks of treatment. Data as mean ± SD. PA, prealbumin; ALB, albumin; TRF, transferrin; Hb, hemoglobin.

Nutrition-related indicators were observed before treatment and after 8 weeks of treatment, and the results displayed that baseline levels of PA, ALB, TRF, and Hb showed no significant differences in the two groups (*P* > 0.05), demonstrating their comparability. After treatment, these nutritional markers improved significantly in both groups, with the study group showing higher levels than the control group (*P* < 0.05) (**Figure 4**).

### Oxidative stress status

**Figure 5:**
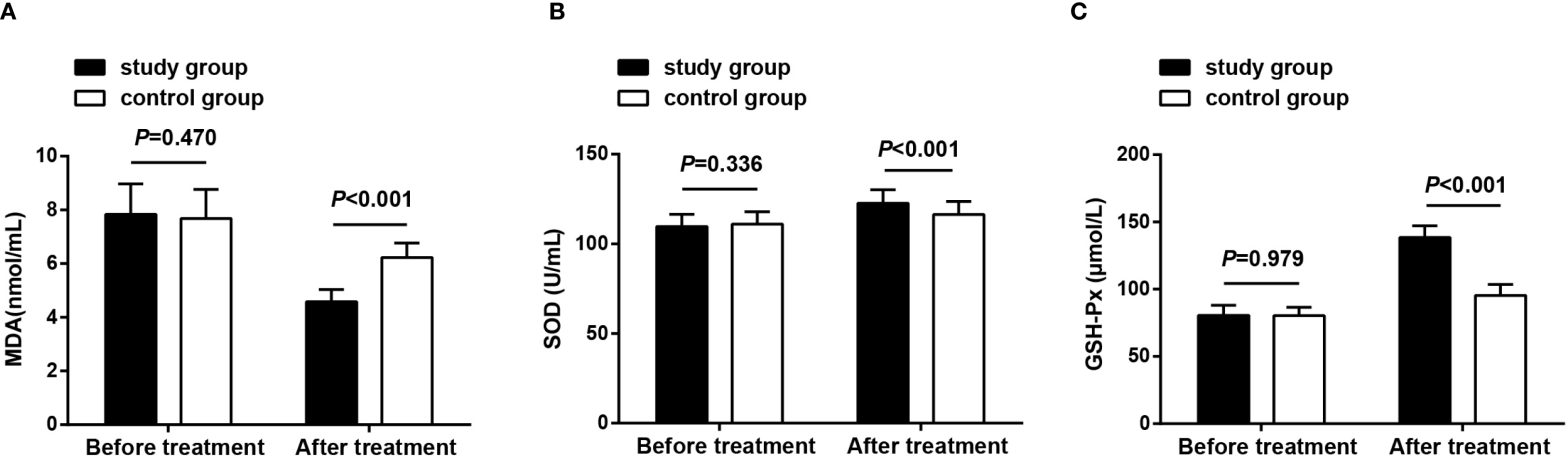
Oxidative stress status before treatment and after 8 weeks of treatment. **(A)** MDA levels in both groups before treatment and after 8 weeks of treatment; **(B)** SOD levels in both groups before treatment and after 8 weeks of treatment; **(C)** GSH-Px levels in both groups before treatment and after 8 weeks of treatment. Data as mean ± SD. MDA, malondialdehyde; SOD, superoxide dismutase; GSH-Px, glutathione peroxidase.

Oxidative stress-related indicators were observed before treatment and after 8 weeks of treatment, and the results revealed that before treatment, no significant differences were observed in serum MDA, SOD, and GSH-Px levels between both groups (*P* > 0.05), demonstrating their comparability; after treatment, MDA levels decreased, while SOD and GSH-Px levels increased in both groups. The study group exhibited lower MDA levels and higher SOD and GSH-Px levels compared to the control group (*P* < 0.05) (**Figure 5**).

### Occurrence of adverse reactions

There was no statistically significant difference in the incidence of adverse reactions between the two groups (*P* > 0.05). All adverse reactions were mild and did not interfere with the subsequent treatment (**Table 3**).

**Table 3:** Comparison of the occurrence of adverse reactions in two groups.

Adverse reactions	The control group (n = 45)	The study group (n = 47)	*P* value
Dizziness and headache	2 (4.44%)	3 (6.38%)	–
Nausea and vomiting	1 (2.22%)	2 (4.26%)	–
Skin itching	2 (4.44%)	3 (6.38%)	–
Abdominal pain and bloating	1 (2.22%)	1 (2.13%)	–
Total incidence	6 (13.33%)	9 (19.15%)	0.450

Data as n(%).

## Discussion

Tubulointerstitial disease, glomerulonephritis, pyelonephritis, and kidney stones are common causes of CKD. Dietary modifications and the avoidance of nephrotoxic drugs are critical measures for slowing the progression of the disease (16). CRF is characterized by the impairment and eventual failure of renal excretory function, resulting in metabolic imbalances due to the retention and accumulation of nitrogenous wastes and other harmful substances (17). Dialysis therapy and pharmacologic interventions remain the cornerstone treatments for CRF. In this paper, we demonstrated the favorable efficacy of combining BLC and low-calcium PDS in managing PD patients with CRF.

As reported in recent research, BLC (18) has been increasingly applied in the treatment of nephropathy. It has demonstrated the ability to modulate immune function, restore renal tubular epithelial cells, prevent tubular atrophy, decrease renal injury, and enhance overall renal function (9). Evidence also suggests that BLC treatment for patients with nephrotic syndrome offers higher clinical efficacy in ameliorating renal function and a lower incidence of adverse effects in contrast with conventional treatment (19). Furthermore, BLC appears to be a cost-effective treatment option for patients with diabetic nephropathy (20). In studies evaluating the combination of BLC with losartan for the treatment of diabetic glomerulosclerosis (DG), results revealed enhanced treatment efficacy. The combination improved blood and urine biochemical indices, renal function, and clinical symptoms, while reducing oxidative stress and the microinflammatory state. It also slowed disease progression without increasing adverse effects (21). These findings align with the results of the present study. Additionally, evidence supports the efficacy and safety of BLC in combination with compound α-ketoacetic acid (KA) tablets for patients with stage 3 CKD. The combination alleviated symptoms such as malaise, anorexia, bad breath, nausea, itching, and edema, while improving renal function (9). These outcomes unearth that BLC, whether used alone or in combination with other treatments, can provide significant therapeutic benefits in nephropathy management.

Moreover, for patients undergoing PD, low-calcium dialysate is recommended as the preferred option to more effectively manage renal osteodystrophy (22). PD is a widely available renal replacement therapy that offers several advantages over central hemodialysis, including greater flexibility in scheduling and the gentle removal of salt and water without significant hemodynamic changes. This sustained and gradual removal of solutes and fluids contributes to better preservation of RRF (23). The preservation of RRF is strongly related to improved survival rates in patients with ESRD. RRF is also linked to enhanced volume and nutritional status, decreased erythropoietin requirements, and a lower incidence of peritonitis in patients with PD. As such, maintenance of RRF is a critical endpoint in the treatment of PD patients (24). Evidence from the literature reveals that incremental PD, defined as less than a full-dose PD prescription, is associated with better preservation of RRF, reduced peritoneal glucose exposure, and a lower risk of peritonitis (25). For patients undergoing continuous ambulatory PD, the use of dialysate with a calcium concentration of 1.25 mmol/L allows for several benefits. These include reduced reliance on aluminum-containing phosphate binders, increased doses of calcium carbonate, and the safe administration of pulsed oral 1α-hydroxyvitamin D3 without inducing severe hypercalcemia. Following an initial increase, PTH levels stabilize within normal or near-normal ranges. Long-term outcomes demonstrate that this approach effectively inhibits the progression of secondary hyperparathyroidism (26). Additionally, low-calcium PDS is commonly used in continuous ambulatory PD to reduce the risk of serum hypercalcemia in patients taking calcium salts as phosphate binders (27). For patients undergoing PD, low-calcium dialysate remains the first choice for optimizing the management of renal osteodystrophy (22).

The results of this study demonstrate that both the control group (treated with low-calcium PDS) and the study group (treated with BLC combined with low-calcium PDS) showed significant improvements after treatment compared to their pre-treatment levels. Both groups exhibited reduced RRF and lower inflammatory markers (TNF-α, CRP, IL-6), as well as higher levels of nutritional markers (serum PA, ALB, TRF, and Hb). Additionally, oxidative stress markers improved, with lower levels of MDA and higher levels of SOD and GSH-Px. Adverse reactions in both groups were mild and did not interfere with subsequent treatment, indicating that low-calcium PDS alone provides certain therapeutic benefits for patients with CRF undergoing PD. These benefits include improvements in RRF, microinflammatory status, nutritional status, oxidative stress status, and a favorable safety profile. However, the study group demonstrated significantly greater improvements compared to the control group. Specifically, the study group exhibited a smaller decline in RRF. Furthermore, more pronounced enhancements were observed in the microinflammatory status, nutritional markers, and oxidative stress parameters. These findings suggest that the combination of BLC and low-calcium PDS provides superior efficacy compared to low-calcium PDS alone for CRF patients undergoing PD. This combination not only better preserves RRF but also delivers more substantial improvements in inflammation, nutrition, and oxidative stress, making it a more effective therapeutic option.

From a pharmacological perspective, the active ingredients in BLC, such as cordycepin, may collectively contribute to the aforementioned synergistic effects through their favorable pharmacokinetic properties (e.g., good oral absorption) and multiple pharmacodynamic mechanisms. Studies have demonstrated that cordycepin can inhibit key inflammatory signaling pathways like NF-κB, thereby reducing the transcription and release of cytokines such as TNF-α and IL-6, leading to a more potent alleviation of the microinflammatory state (28, 29). Additionally, it can mitigate oxidative stress-induced damage to residual nephrons by enhancing the activities of enzymes like SOD and GSH-Px (30–32). Furthermore, its effects on improving protein and energy metabolism (33, 34) also contribute to enhanced nutritional indicators. These mechanisms complement the local effects of low-calcium peritoneal dialysis solution, providing evidence for sustained renal protection during the 8-week treatment period and supporting the observed dose-response relationship and superiority of the combined therapy from a biological mechanistic standpoint.

## Conclusion

Overall, BLC in combination with low-calcium PDS has demonstrated promising results in patients with CRF undergoing PD. This combination therapy effectively slows the decline in RRF and improves the microinflammatory state, nutritional status, and oxidative stress status, all while maintaining a favorable safety profile and not increasing the incidence of adverse effects. Historically, treatments for CRF patients undergoing PD have primarily focused on either PD or pharmacological therapies independently. This study introduces an innovative approach by combining BLC with low-calcium PDS to evaluate their synergistic effects on RRF and microinflammatory status in these patients. Despite its promising findings, the study has several limitations. Firstly, this study is a single-center trial, and there remain considerable issues regarding the reliability of the results and the feasibility of the experiment. Secondly, the relatively short observation period and inadequate follow-up duration restrict the ability to fully evaluate the long-term efficacy and safety of this combined therapy. Additionally, this study lacks follow-up on other important clinical outcomes, such as survival rate and quality of life. Future research should address these limitations by incorporating a larger sample size and conducting long-term follow-ups to provide a more comprehensive evaluation of the combined therapy’s efficacy and safety. Meanwhile, expanded future research to explore the relevant mechanisms underlying the actions of BLC is imperative. Optimizing study designs and including additional clinical outcomes, such as survival rate and quality of life, will further refine our understanding of the benefits of BLC combined with low-calcium PDS in this patient population.

## Data Availability

The original contributions presented in the study are included in the article/supplementary material. Further inquiries can be directed to the corresponding author.
